# Rational and practical exfoliation of graphite using well-defined poly(3-hexylthiophene) for the preparation of conductive polymer/graphene composite

**DOI:** 10.1038/srep39937

**Published:** 2017-01-06

**Authors:** Hiroki Iguchi, Chisato Higashi, Yuichi Funasaki, Keisuke Fujita, Atsunori Mori, Akira Nakasuga, Tatsuo Maruyama

**Affiliations:** 1 Department of Chemical Science and Engineering, Graduate School of Engineering, Kobe University, 1-1 Rokkodaicho, Nada-ku, Kobe 657-8501, Japan; 2 Sekisui Chemical Co., Ltd., 2-1 Hyakuyama, Shimamoto-cho, Mishima-gun, Osaka 618-0021, Japan

## Abstract

Processing and manipulation of highly conductive pristine graphene in large quantities are still major challenges in the practical application of graphene for electric device. In the present study, we report the liquid-phase exfoliation of graphite in toluene using well-defined poly(3-hexylthiophene) (P3HT) to produce a P3HT/graphene composite. We synthesize and use regioregular P3HT with controlled molecular weights as conductive dispersants for graphene. Simple ultrasonication of graphite flakes with the P3HT successfully produces single-layer and few-layer graphene sheets dispersed in toluene. The produced P3HT/graphene composite can be used as conductive graphene ink, indicating that the P3HT/graphene composite has high electrical conductivity owing to the high conductivity of P3HT and graphene. The P3HT/graphene composite also works as an oxidation-resistant and conductive film for a copper substrate, which is due to the high gas-barrier property of graphene.

Graphene is one of the most interesting nanomaterials owing to its unique properties, such as high electrical conductivity, high mechanical toughness, high gas-barrier performance, and unique optical properties^1^,^2^,^3^. Industrial applications of graphene are expected in a wide range of areas, including electronic materials and medical engineering. In particular, its application in electronics has been highly expected and widely studied. Since the pioneering work on mechanical exfoliation for the production of graphene in 2004^1^, a number of methods for the production of graphene have been proposed: for example, mechanical exfoliation^1^, chemical vapor deposition (CVD)^4^,^5^, heat decomposition^6^, and chemical synthesis^7^. Many of these methods are costly, ineffective, and non-scalable. ‘Liquid-phase exfoliation’ of graphite is one of the most inexpensively scalable approaches. However, graphene usually aggregates in solvents because of the strong hydrophobic interaction and van der Waals force between graphene sheets. The aggregation causes a loss of favorable properties and increases the difficulty for the practical manipulation of graphene. Liquid-phase exfoliation is divided into chemical and physical processes. Ever since chemically exfoliated graphene (e.g., graphene oxide, acidified graphene, and reduced graphene oxide) was shown to have a number of structural defects^8^,^9^, recent studies have focused on a physical process, especially on pristine graphene produced by the liquid-phase exfoliation of graphite. Various types of dispersants (surfactants, polymers, ionic liquids, proteins, and nanotubes) have been proposed for the direct production of pristine graphene from graphite^10^,^11^,^12^,^13^,^14^,^15^,^16^. Many of these dispersants, however, decrease the electrical conductivity of graphene because they disrupt the extended aromatic conjugated systems and cover the graphene with nonconductive molecules^16^,^17^.

Poly(3-hexylthiophene) (P3HT) has attracted attention in fields such as material science and electronics owing to its high electrical conductivity and its processability^18^. Although π–π interactions between P3HT and carbon nanomaterials (carbon nanotubes and graphene oxides) have been observed^19^,^20^,^21^,^22^,^23^,^24^, there is, to the best of our knowledge, no report on the exfoliation of pristine graphite using P3HT to produce a stable dispersion of graphene. Regioregularity of P3HT plays an important role in the electrical conductivity of P3HT^18^. Our group has already succeeded in the regiochemically-controlled synthesis of P3HT having controlled molecular weights^25^,^26^,^27^. In this study, we propose the exfoliation of pristine graphite in toluene using well-defined P3HT as a conductive dispersant to produce pristine graphene without loss of the electrical conductivity (Fig. 1). The present study also demonstrates that the P3HT/graphene composite can be used as conductive ink and an oxidation-resistant film for a metal substrate.

## Methods

### Materials

Graphite flakes and copper-plated divinylbenzene microparticles (CuMPs) were provided by Sekisui Chemical Co., Ltd. (Tokyo, Japan). 2-Bromo-3-hexylthiophene and [1,3-bis(2,6-diisopropylphenyl)imidazol-2-ylidene]triphenylphosphine nickel(II) dichloride (NiCl_2_(PPh_3_)IPr) were purchased from Tokyo Chemical Industries, Ltd. (Tokyo, Japan). 2,2,6,6-Tetramethylpiperidinylmagnesium chloride lithium chloride complex (TMPMgCl·LiCl) solution was purchased from Sigma (St. Louis, MO). Other chemicals were purchased from Wako PureChemical Industries, Ltd. (Osaka, Japan). The water used was high-quality deionized water (Milli-Q water, >15 MΩ cm^−1^) produced using a Milli-Q Advantage A100 system equipped with an Elix UV 3 system (Millipore, Molsheim, France).

### Polymerization of 2-bromo-3-hexylthiophene using a Knochel-Hauser base

P3HT was synthesized by the slightly modified procedure previously reported by us^25^,^26^,^27^. To a THF solution containing 1.0 M TMPMgCl·LiCl (1.56 mmol), 2-bromo-3-hexylthiophene (1.3 mmol, 324 mg) was added dropwise at 25 °C. After stirring at 25 °C for 3 h, THF (20 mL) and NiCl_2_(PPh_3_)IPr (28.3 mg, 0.036 mmol) were successively added. The resulting mixture was stirred at 25 °C for 1 h. Hydrochloric acid (1.0 M, 20 mL) and methanol (20 mL) were added to form a precipitate. The mixture was filtered and the residue was washed with methanol and *n*-hexane repeatedly to obtain a dark purple solid, which was dried under reduced pressure to yield 144 mg of poly(3-hexylthiophene) (P3HT) (66%). Molecular weights and the molecular weight distribution of P3HT were estimated by SEC analysis (eluent: CHCl_3_) using polystyrene as the molecular weight standard. For example, SEC analysis showed *M*_n_ = 6000, and *M*_w_/*M*_n_ = 1.10. The regioregularity was estimated by ^1^H NMR analysis (thienyl-CH_2_- signals) at the δ 2.80 (H-T) and δ 2.60 (H-H) signals (H-T regioregularity = 97%).

Regio-irregular P3HT (H-T regioregularity 77%, Mn 7000 and *M*_*w*_*/M*_*n*_ = 1.6) was synthesized according to a previous report^28^.

### Preparation of graphene dispersion with P3HT

Graphite flakes were ultrasonicated (AsOne Corporation., Ultrasonic cleaner VS-D100, 31 kHz) with P3HT in toluene at 80 °C for 30 min, followed by incubation at 30 °C for 15 min. The concentration of graphite flakes was 0.33 mg mL^−1^. By adding the different quantities of P3HT, the mixed ratios of P3HT/graphite were varied to 0, 0.1, 0.2, 0.5, 0.75, 1.0, and 1.5 wt/wt. Following ultrasonication, the dispersions were centrifuged (Hitachi Koki Co., Ltd., CF15RX) at 1000 × *g* for 20 min to remove the unexfoliated particles and thick flakes of graphite. Then, the supernatant was collected as the graphene dispersion.

### Preparation of P3HT/graphene composite film-paper

P3HT/graphene composite film-papers were prepared by vaccum filtration. Graphene dispersions (3 ml) containing 42 μg graphene, which were prepared using P3HT (*M*_n_ = 6,000) or SDS, were filtrated through filter paper that were 8 mm in diameter and 4 μm of pore size (Kiriyama Grass Co., Ltd.). P3HT/graphene (or SDS/graphene) on the filter paper was dried at 100 °C for 5 min to ensure complete solvent evaporation.

### Preparation of P3HT/graphene-coated CuMPs

The CuMPs were rinsed with H_2_SO_4_ aqueous solution (0.1 M) for 10 min at 25 °C to remove copper oxide from the surfaces. Then, they were rinsed with excess amounts of water and ethanol three times each. P3HT- and P3HT/graphene-coated CuMPs were prepared by dip-coating the rinsed CuMPs (20 mg) in P3HT/graphene dispersion (400 μL) for 1.5 h at 40 °C, followed by the removal of the supernatant by vaccum evaporation overnight to dry the P3HT/graphene-coated CuMPs.

### Sample characterization

UV/Vis absorption spectra of the dispersions (200–900 nm) were recorded using a double-beam spectrophotometer (JASCO, V-770). Since P3HT did not exhibit an absorbance around 660 nm, absorption measurements at 660 nm allowed us to evaluate the concentration of dispersed graphene in toluene.

Raman spectroscopy measurements were performed on a Nano-Raman system WITec alpha300R using a laser excitation at 532 nm (Nd-YAG-neodymium-doped yttrium aluminium garnet type laser) at 3 mW.

TEM images were obtained using a JEOL 2100 F transmission electron microscope operated at 200 kV. A drop of graphene dispersion was placed on a copper grid covered with elastic carbon films manufactured by Okenshoji Co., Ltd. and dried under vaccum at ambient temperature.

AFM images were obtained in a tapping mode using Hitachi Nanonavi E-sweep with cantilevers at 17,000 N m^−1^. SEM observations were performed using a JEOL JSM-7500F scanning electron microscope.

Electrical resistance measurements were performed using a four-point-probe ohmmeter (Mitsubishi Chemical Analytec Co., Ltd. LorestaAX MCP-T370, TFP-prove) for P3HT/graphene composite paper films and a digital multimeter (Custom Co., Ltd. CDM-200D) for CuMPs.

## Results

**Figure 2:**
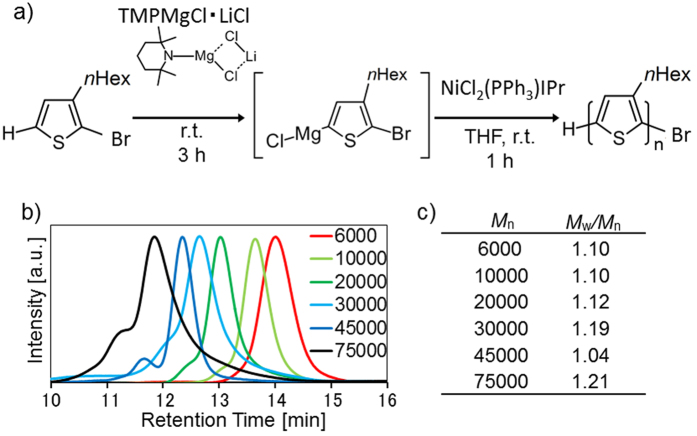
(**a**) Polymerization of 2-bromo-3-hexylthiophene using the Knochel-Hauser base and a nickel catalyst. (**b**) Size-exclusion chromatography (SEC) profiles of synthesized P3HT with different *M*_n_. (**c**) Number-average molecular weights and polydispersity indices (*M*_w_*/M*_n_) of synthesized P3HT.

Regioregular P3HT with controlled molecular weights were synthesized according to our previous report (Fig. 2a)^25^. Figure 2b shows the elution profiles of the synthesized P3HT from size-exclusion chromatography (SEC). The SEC profiles reveal that the synthesized P3HT had *M*_n_ ranging from 6,000 to 75,000 with a very narrow distribution of molecular weights (*M*_w_*/M*_n_ was from 1.04 to 1.21, Fig. 2c), which agrees with our previous reports^25^,^26^,^27^.

**Figure 3:**
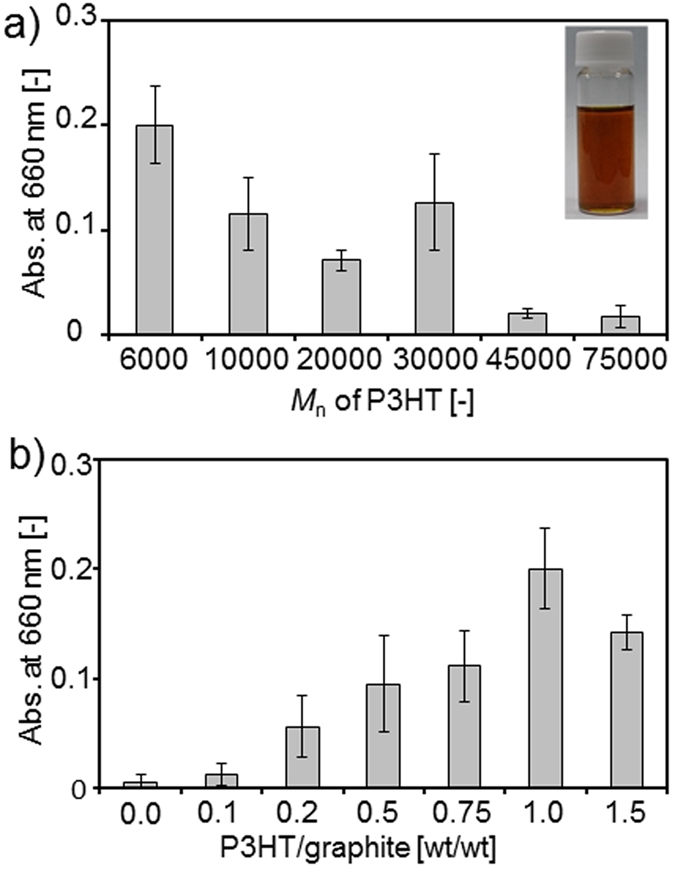
Absorbance at 660 nm of graphene dispersions in toluene. (**a**) Effect of different *M*_n_ of P3HT (the P3HT/graphite ratio was 1.0) and (**b**) effect of different mixed ratios of P3HT (*M*_n_ = 6,000)/graphite on the absorbance. The concentration of graphite flakes was 0.33 mg mL^−1^. The inset represents a photo of a P3HT (*M*_n_ = 6000)/graphene dispersion.

Graphite flakes (Fig. S1, Supporting Information) were mixed with P3HT in toluene and ultrasonicated at 80 °C for 30 min. After centrifugation, the resultant toluene solution containing P3HT and graphene was dark brown (inset of Fig. 3a). Figure S2 shows the absorption spectra of the P3HT and P3HT/graphene solutions. The P3HT/graphene solution exhibits a plateau absorbance over 600 nm, which was not observed in the P3HT solution. These results indicate the exfoliation of graphite by P3HT in toluene to produce the P3HT/graphene dispersion^12^.

We evaluated the effect of the *M*_n_ of P3HT on the dispersing graphene by measuring the absorbance of the P3HT/graphene dispersion at 660 nm^12^. Figure 3a reveals that low-molecular-weight P3HT (*M*_n_ = 6,000) was the most effective in dispersing graphene. P3HT with molecular weights of more than 40,000 resulted in low graphene concentrations in the dispersion, which might be related to the low solubility of the high-molecular-weight P3HT in toluene^18^,^29^.

The effect of the P3HT/graphite ratio on dispersing graphene was also investigated (Fig. 3b). As the ratio was increased up to 1.0, the dispersing ability of P3HT increased. At a ratio of 1.5, the dispersing ability slightly decreased. When P3HT with *M*_n_ 6,000 was used and the P3HT/graphite was 1.0, the graphene concentration in toluene was 14 μg mL^−1^, in which the yield of graphene was 3.5%.

We also used relatively regio-irregular P3HT (H-T regioregularity 77%, *M*_*n*_ 7000 and *M*_w_/*M*_n_ = 1.6) at a P3HT/graphite ratio of 1.0 to exfoliate graphite and to disperse graphene in toluene. Interestingly, there observed negligible absorbance at 660 nm (Fig. S3), meaning almost no exfoliation of graphite and no graphene dispersion. The twisted structure of the regio-irregular P3HT might inhibit its intercalation into graphite and might be unable to form a stable composite with a graphene sheet. These results proved the importance of the P3HT regioregularity in the exfoliation of graphite and in the dispersion of graphene. The following experiments were carried out using regioregular P3HT with *M*_n_ of 6,000 and at a P3HT/graphite ratio of 1.0.

**Figure 4:**
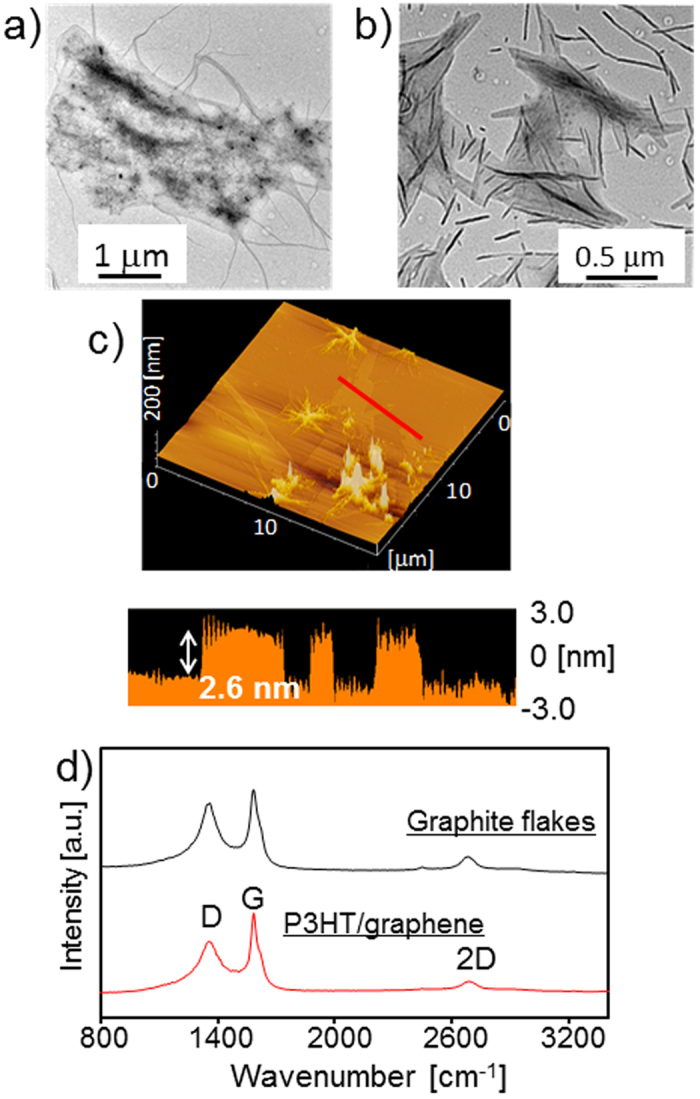
(**a**,**b**) TEM images of graphene sheets with P3HT (*M*_n_ = 6,000). (**c**) AFM image of P3HT/graphene deposited on a mica substrate with a height profile along the red line. (**d**) Raman spectra of graphite flakes and P3HT/graphene composite. P3HT (*M*_n_ = 6,000) was used for preparing the P3HT/graphene complex.

The P3HT/graphene dispersion was characterized using a transmission electron microscope (TEM), an atomic force microscope (AFM), and Raman spectroscopy. TEM images of the P3HT/graphene dispersion are shown in Fig. 4a and b. Large graphene sheets with lateral dimensions of 0.1–2 μm were observed. Tri-layer and single-layer graphene were observed by considering the edge of the graphene in Fig. 4a and b, respectively. Nanowires of P3HT were also observed, which is consistent with a previous report^21^. Figure 4c shows the AFM image of the P3HT/graphene dispersion deposited on a mica substrate and the height profile along the red line in the figure revealed that the thickness of P3HT/graphene was 2.6 nm, which corresponded to tri-layer graphene with P3HT^15^. These observations clearly demonstrate that graphite flakes were exfoliated by P3HT to produce single-layer and few-layer graphene sheets.

The above investigations suggest that P3HT works as a dispersant for graphene in toluene. Defects in graphene often occur during the exfoliation of graphite and dispersion of graphene in solvents^30^,^31^. We measured the Raman spectra of graphite flakes and the dry P3HT/graphene composite. Figure 4d shows the D band (1360 cm^−1^), G band (1584 cm^−1^), and 2D band (2680 cm^−1^) of the analytes^32^,^33^. The D/G band intensity ratio indicates the degree of structural defects in graphene^32^. The dry P3HT/graphene composite showed an *I*_D_/*I*_G_ ratio of 0.63, which was almost the same as that of the graphite flakes. These measurements indicate that defects in the graphene sheet did not occur during the P3HT-induced exfoliation of the graphite and graphene dispersion.

**Figure 5:**
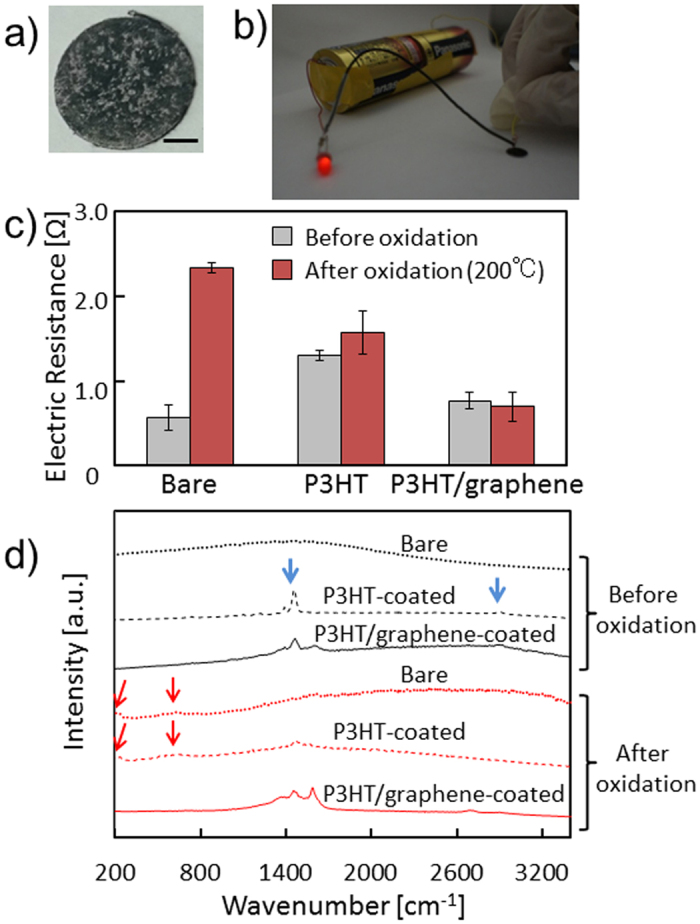
(**a**) P3HT/graphene composite filtrated and collected on filter paper. Scale bar represents 2 mm. (**b**) Photograph of a lit LED assembled with a P3HT/graphene composite and a battery. (**c**) Electrical resistance and photographs of bare CuMPs, P3HT-coated CuMPs, and P3HT/graphene-coated CuMPs before and after oxidation. (**d**) Raman spectra of bare CuMPs, P3HT-coated CuMPs, and P3HT/graphene-coated CuMPs before (black) and after (red) oxidation treatment.

To investigate the electrical property of the P3HT/graphene composite, P3HT/graphene and sodium dodecyl sulfate (SDS)/graphene composites on filter paper were prepared by vacuum filtration of the graphene dispersion (Fig. 5a)^34^. The sheet resistance of the P3HT/graphene composite was 1.8 ± 0.5 kΩsq^−1^, which was much smaller than that of the SDS/graphene composite (31.6 ± 3.0 kΩsq^−1^). The resistance of the P3HT/graphene-composite was comparable with those of graphene films with other dispersants^11^,^15^,^35^, of CVD-grown graphene^36^,^37^ and of commercially available graphene^38^. We assembled the P3HT/graphene composite with a circuit and succeeded in energization (LED lighting in Fig. 5b). The remarkably low resistance of the P3HT/graphene composite arose from the high conductivity of P3HT. These investigations proved the great potential of the P3HT/graphene dispersion for use as conductive ink^12^,^35^,^39^,^40^.

One of the unique properties of graphene is its molecular barrier property, which prevents gas permeation^3^,^41^,^42^,^43^. We anticipated that the P3HT/graphene composite can be used for the anti-oxidation of oxidizable metal substrates in electronics. Gold- and silver-coated substrates are widely used in electronics and in electrical appliances owing to their high conductivity and high stability in the atmosphere. Although the replacement of these precious metals with base metals (e.g., Cu and Ni) is very cost-effective, a major drawback in the electric industry is that base metals are readily oxidized in the atmosphere to produce non-conductive metal oxides. We prepared Cu-microparticles (CuMPs, 0.2 mm in diameter) coated with the P3HT/graphene composite by dip-coating to evaluate the anti-oxidation property of the P3HT/graphene coating.

Figure 5c shows that the electrical resistance of bare CuMPs was 0.6 ± 0.2 Ω and that the oxidation treatment increased the resistance by more than 4 times (2.3 ± 0.1 Ω). The P3HT-coated CuMPs exhibited a resistance of 1.3 ± 0.1 Ω before oxidation and 1.6 ± 0.3 Ω after oxidation. Although the sole P3HT coating also suppressed the oxidation to a certain extent, the intrinsic resistance of the P3HT-coated CuMPs was relatively high. The P3HT/graphene-coated CuMPs exhibited a resistance of 0.8 ± 0.2 Ω before oxidation, which was comparable with that of the bare CuMPs. After the oxidation treatment, the resistance of the P3HT/graphene-coated CuMPs remained at 0.7 ± 0.2 Ω, which was as low as that of the bare CuMPs. These results show that the P3HT/graphene coating prevented Cu oxidation and maintained the intrinsic resistance of the CuMPs.

To verify the coatings on the CuMPs, we recorded the Raman spectra for the surfaces of the bare and coated CuMPs before and after the oxidation treatment. Figure 5d shows small peaks at 600 and 220 cm^−1^ (red arrows in Fig. 5d) for the bare CuMPs and the P3HT-coated CuMPs after the oxidation treatment, which result from the production of CuO and Cu_2_O. The observed peaks for P3HT are at 2,900 and 1,500 cm^−1^ (blue arrows in Fig. 5d) in the spectra of the P3HT-coated CuMPs. These results suggest successful P3HT coating of CuMPs and Cu oxidation for the P3HT-coated CuMPs by the oxidation treatment. In the spectrum of the P3HT/graphene-coated CuMPs, no peaks for copper oxide were observed while peaks for graphene (D, G, and 2D band) and peaks for P3HT were observed, showing the inhibition of Cu oxidation and the successful P3HT/graphene coating. The inhibition of Cu oxidation is proposed to arise from the high gas-barrier property of graphene. It should be noted that a peak at 1600 cm^−1^ was assigned as a G peak of graphene^32^,^33^.

Figure S4 (Supporting Information) shows the FE-SEM images of bare, and P3HT- and P3HT/graphene-coated CuMPs. A wrinkled morphology was observed on the surface of the P3HT-coated CuMPs in the magnified image (right photographs of Fig. S4b, Supporting Information). The P3HT/graphene-coated CuMPs also had a wrinkled surface, which was more obvious in the left of Fig. S4c than in Fig. S4b (Supporting Information). These wrinkled morphologies provided evidence for the coatings of P3HT and the P3HT/graphene on CuMPs.

## Discussion

In this study, we first succeeded in the exfoliation of pristine graphite using well-defined P3HT to stably disperse graphene in toluene. In particular, regioregular P3HT with *M*_n_ of 6,000 was found to effectively exfoliate graphite flakes to produce graphene and to stably disperse graphene in toluene, in which π–π interaction between P3HT and graphene would play an important role in the formation of the P3HT/graphene composite. The regioregularity of P3HT was also important for the exfoliation of graphite and the dispersion of graphene. The P3HT/graphene composite showed high electrical conductivity owing to the high conductivity of P3HT and graphene, which proposes the potential application of the P3HT/graphene dispersion as conductive graphene ink. The investigations on the P3HT/graphene coating interestingly demonstrate that the P3HT/graphene composite can be used as an oxidation-resistant and conductive film for a copper substrate. Liquid-phase exfoliation of pristine graphite and stable dispersion of graphene, without losing the properties of graphene, extend the practical applications of graphene. Although there were, to the best of our knowledge, only a few reports that proposed the application of graphene utilizing both its electrical conductivity and gas-barrier property^36^,^44^,^45^, the present study clearly demonstrates the high potential of the P3HT/graphene composite as a gas barrier and conductive nanomaterial that is scalable and processable.

## Additional Information

**How to cite this article:** Iguchi, H. *et al*. Rational and practical exfoliation of graphite using well-defined poly(3-hexylthiophene) for the preparation of conductive polymer/graphene composite. *Sci. Rep.* **7**, 39937; doi: 10.1038/srep39937 (2017).

**Publisher's note:** Springer Nature remains neutral with regard to jurisdictional claims in published maps and institutional affiliations.

## Supplementary Material

Supplementary Information

## Figures and Tables

**Figure 1:**
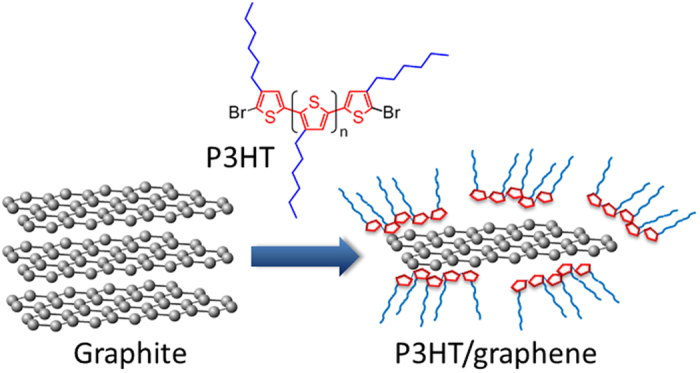
Schematic illustration of the exfoliation of graphite and the graphene dispersion using poly(3-hexylthiophene).
