# Supplementary material for: Rational and practical exfoliation of graphite using well-defined poly(3-hexylthiophene) for the preparation of conductive polymer/graphene composite
Source: Sci Rep. 2017 Jan 6;7:39937. doi: 10.1038/srep39937 (PMC5216383; doi:10.1038/srep39937)
Supplement: Supplementary Information [file srep39937-s1.pdf]

Supplementary Information

Rational and practical exfoliation of graphite using well-defined poly(3-hexylthiophene) for the preparation of conductive polymer/graphene composite

Hiroki Iguchi,[1] Chisato Higashi,[1] Yuichi Funasaki,[1] Keisuke Fujita,[1] Atsunori Mori,[1] Akira Nakasuga,[2] and Tatsuo Maruyama[1,]*

[1]Department of Chemical Science and Engineering, Graduate School of Engineering, Kobe University, 1-1 Rokkodaicho, Nada-ku, Kobe 657-8501, Japan

[2]Sekisui Chemical Co., Ltd., 2-1 Hyakuyama, Shimamoto-cho, Mishima-gun, Osaka 618-0021, Japan

Correspondence to tmarutcm@crystal.kobe-u.ac.jp

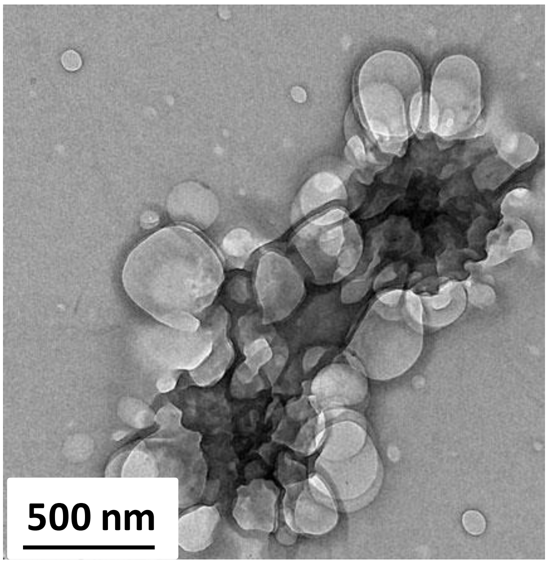

Figure S1. TEM image of graphite flakes.

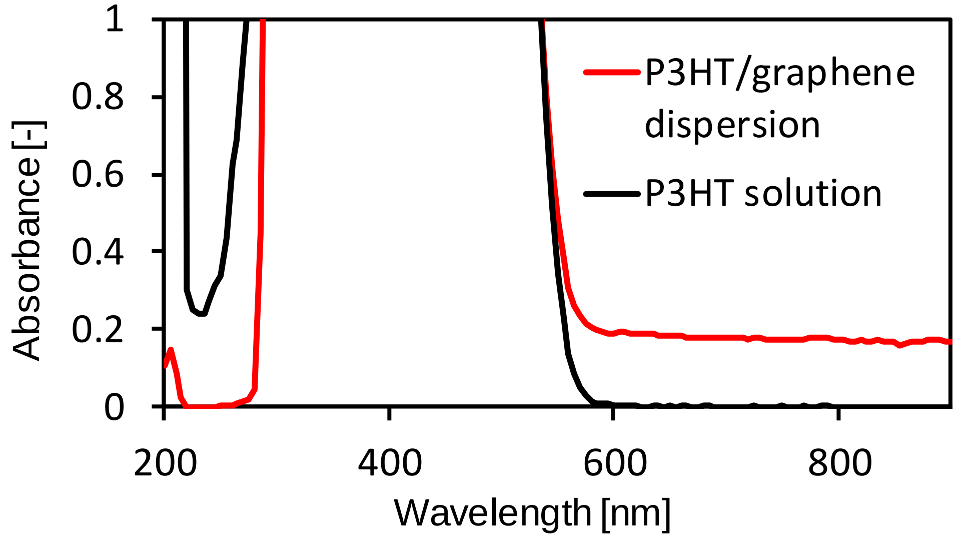

Figure S2. UV/Vis absorption spectra of the P3HT toluene solution and P3HT/graphene dispersion in toluene. The concentration of P3HT was 0.33 mg mL^{-1} and that of graphite was 0.33 mg mL^{-1}.

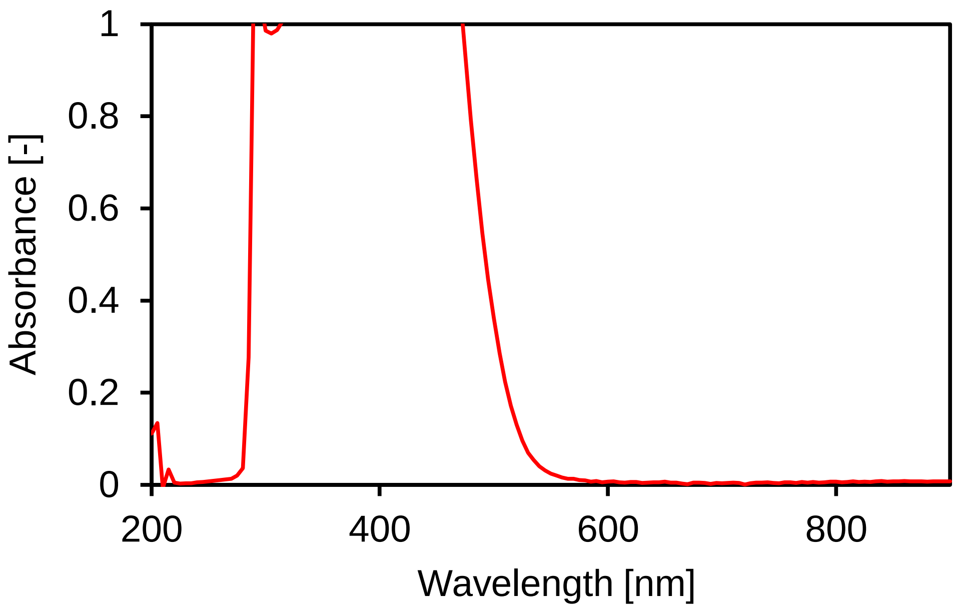

Figure S3 UV/Vis absorption spectra of toluene solutions containing regio-irregular P3HT and regio-irregular P3HT/graphene dispersion. The concentration of regio-irregular P3HT was 0.33 mg mL^{-1} and that of graphite was 0.33 mg mL^{-1}. H-T regioregularity of the regio-irregular P3HT was 77 %. M_n = 7000 and M_w/M_n = 1.6.

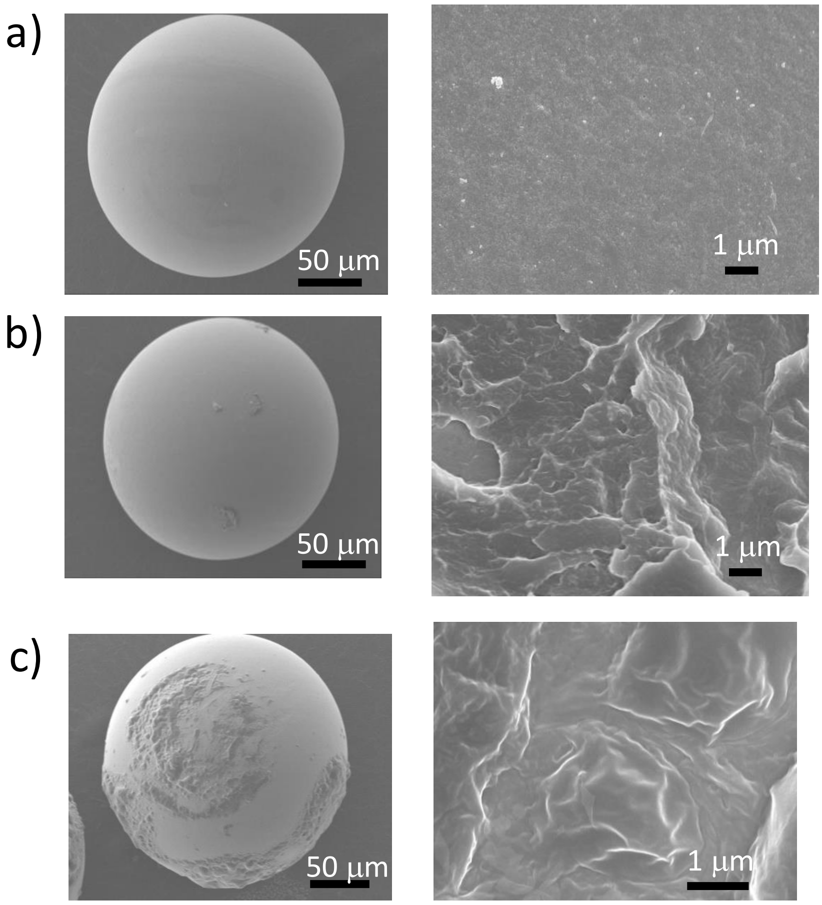

Figure S4. SEM images of (a) bare CuMPs, (b) P3HT-coated CuMPs and (c) P3HT/graphene-coated CuMPs.
